# Autologous replacement of the head of the radius—proximal fibula versus second metatarsal base: an anatomic feasibility study

**DOI:** 10.1007/s00402-022-04460-y

**Published:** 2022-05-09

**Authors:** Sebastian Farr, Julian Augustin, Sebastian Röhrich, Martina Felbermeier, Lena Hirtler

**Affiliations:** 1 Department of Pediatric Orthopaedics and Foot and Ankle Surgery, Orthopaedic Hospital Speising, Vienna, Austria; 2 Krankenhaus der Barmherzigen Schwestern Ried, Ried, Austria; 3 Department of Biomedical Imaging and Image-Guided Therapy, Medical University of Vienna, Vienna, Austria; 4 Center for Anatomy and Cell Biology, Medical University of Vienna, Vienna, Austria

**Keywords:** Radial head, Radial head fracture, Osteoarthritis, Radial head dislocation, Elbow prosthesis, Fibular autograft, Second metatarsal

## Abstract

**Introduction:**

This study investigated the anatomic feasibility of a new surgical therapy option for radial head arthrosis using an autologous vascularized bone graft of the second metatarsal and proximal fibula to recreate the proximal radiohumeral joint.

**Materials and methods:**

Upper and lower extremities of eleven body donors were evaluated using CT prior to anatomic dissection. Several distinct anatomic parameters were measured on the ipsi- and contralateral radial and fibular head and the second metatarsal base: *bone diameter, articular surface diameter, head height, metaphyseal (neck) diameter*, *articular surface radius, total articular surface area*, and *angulation of the articular surfaces (facet)*. Each dissection phase was photographed in a standardized fashion and all measurements were repeated by direct caliper-measurements.

**Results:**

When comparing the proximal radius and fibula to search for anatomic similarities, similar values were found in the maximum *articular surface diameter* and minimum and maximum measures of the *neck diameter*. Comparing the proximal radius and the second metatarsal, statistically similar values were found in the maximum *neck diameter* performing direct measurements and CT evaluation, the maximum *head diameter* in CT evaluation and the *articular facet angulation*.

**Conclusions:**

Neither the proximal fibula nor the base of the second metatarsal are ideal bone grafts for replacement of the head of the radius. The base of the second metatarsal might be a bit more suitable as a potential donor since the angulation of the proximal articular facet is similar to that of the radius.

**Level of evidence:**

Level IV, anatomic study.

## Introduction

Proximal radius fractures in children and adolescents account for around 4–21% of all pediatric elbow injuries (1, 2). Although pediatric radial head/neck fracture treatment can generally be considered successful if initiated promptly, delayed or missed treatment can cause significant morbidity due to the precarious vascularization of the head of the radius and the associated potential for necrosis and remodeling defects (3–5). In this regard, severe proximal radius malalignment may cause progressive radial head dislocation and disruption of the proximal radioulnar joint integrity (6). The head of the radius eventually loses its native concavity and degenerates over time. Thus, the causes for pediatric radial head degeneration and early arthrosis are mainly posttraumatic reasons as well as—more rarely—avascular necrosis (7).

While prosthetic treatment of radial head arthrosis in adults is an established procedure, no such opportunity exists in the pediatric age group (8). Secondary corrective osteotomy techniques are usually effective in reliably realigning the proximal radius since the head of the radius has usually lost its anatomic orientation and congruency. Hence, radial head resection might be the only solution to reduce pain and improve function in this patient cohort (9–12). However, such procedure is not without risks due to the potential occurrence of lateral elbow instability and longitudinal forearm instability (12, 13). The latter may cause a vicious circle of consecutive ulnocarpal impaction syndrome with a need of ulnar shortening procedures due to the proximal radial migration (14). To prevent such occurrence, multiple interposition arthroplasty techniques have already been described for the pediatric population (9, 11).

Given the above-mentioned circumstances and obstacles, native radial head replacement with autologous tissue would be desirable for children, adolescents and young adults. This, however, is a quite difficult task to fulfill, as the elbow joint with its three articular parts is a quite complex joint. Only the interaction between humerus and ulna, humerus and radius as well as radius and ulna allow the unhampered movability and stability of the joint. In this, the morphology of the humeroradial and radioulnar joint surfaces and thus of the proximal radius itself play a large role. The size and the orientation of the superior articular surface of the head of the radius are important for its articulation with the humeral capitulum, the articular surface of the radial circumference and the size of the radial neck are important for its articulation with the radial notch of the ulna and the interaction with the annular ligament of the radius (1, 15–18). Thus, the donor bone should not only involve a bone whose function is sufficiently compensated biomechanically, but which also adequately accomplishes the required tasks in the complex elbow joint.

Based on these anatomic considerations, we hypothesized that the second metatarsal bone and the fibular head would be suitable donors for a replacement of the proximal radius. This study thus investigated the feasibility of a new surgical therapy option using an autologous vascularized bone graft of the second metatarsal and proximal fibula to recreate the radiohumeral joint. Anatomic features and landmarks were evaluated to determine whether these bones would be potential donors for such degenerative indications.

## Materials and methods

For the purpose of this study, specimens from eleven body donors were used. The donors gave their consent during life for their body to be used for scientific and teaching purposes to the Center for Anatomy and Cell Biology of the Medical University of Vienna. Additionally, the study was approved by the ethical committee of the Medical University of Vienna (1314/2017). Of these eleven body donors, both upper and lower extremities were included, resulting in a total of 22 upper and lower extremities, respectively. The specimens were used fresh and were only stored at 4 °C prior to imaging and dissection. Inclusion criteria for this study were the absence of any signs of previous pathologies (e.g., injuries, tumors), previous surgery (e.g., endoprosthetic replacement, plating), advanced osteoarthritis of the joint and congenital malformations in the specimens.

### Evaluation

First, CT evaluation was performed on the entire upper and lower limbs prior to anatomic dissection to preserve anatomic distances in and around the joint area. All CT scans were conducted on the same scanner (Siemens SOMATOM FORCE; Siemens Healthcare AG, Erlangen, Germany) with a slice thickness of 1 mm, 80 kV and activated tube current modulation. A sharp bone kernel (Br69d) was used for reconstruction. The radius and fibula were placed longitudinally along the *Z*-axis of the scanner. For the second metatarsal bone, the foot was fixed in a 90°-angle to the *Z*-axis. Multiplanar reconstructions were aligned in a 90°-angle to and along the long axis of the respective bones. Morphological measurements were taken in accordance with the measurements on anatomical specimens. For CT evaluations, Osirix^®^ (www.osirix-viewer.com) was used.

**Fig. 1:**
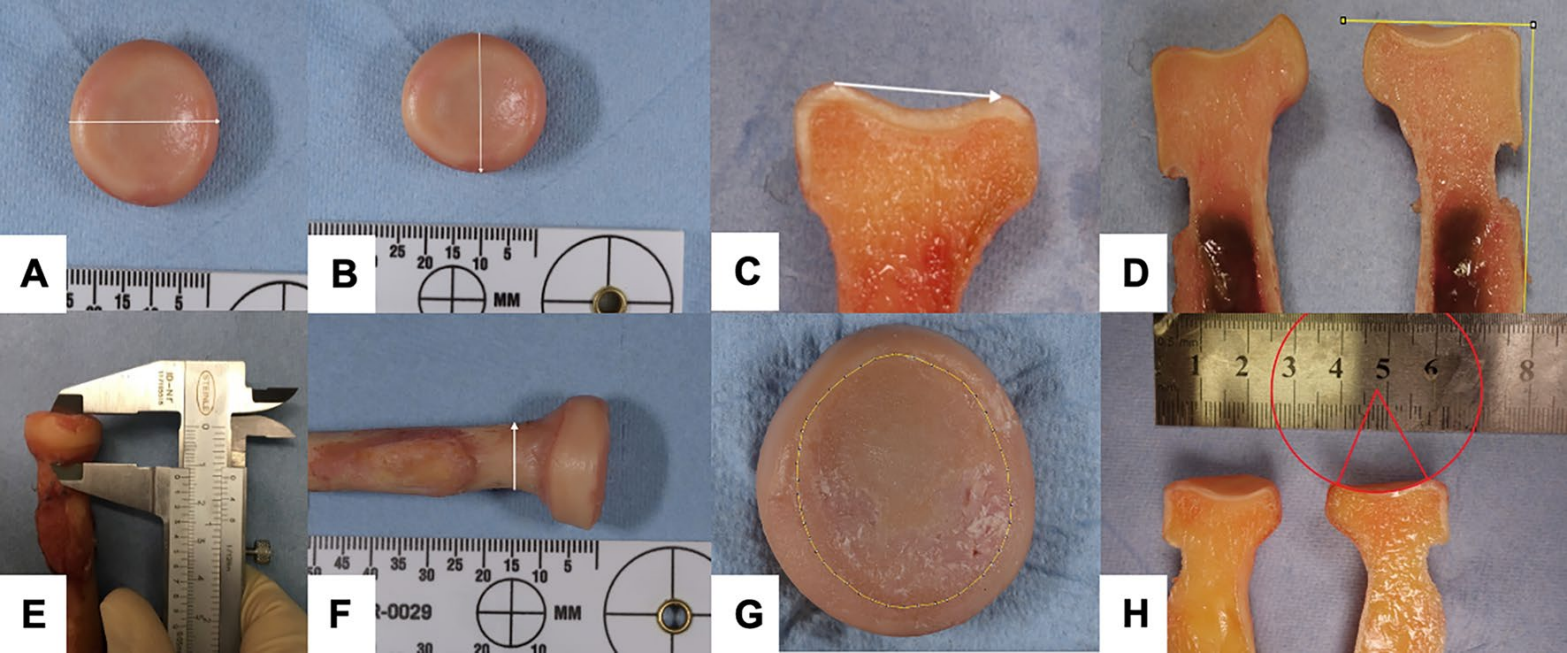
The anatomic radial head measurements are shown: bone diameter (**A**, **B**), articular surface diameter (**C**), angulation of the articular surface (**D**), head height (**E**), neck diameter (**F**), articular surface area (**G**), and articular surface radius (**H**)

**Fig. 2:**
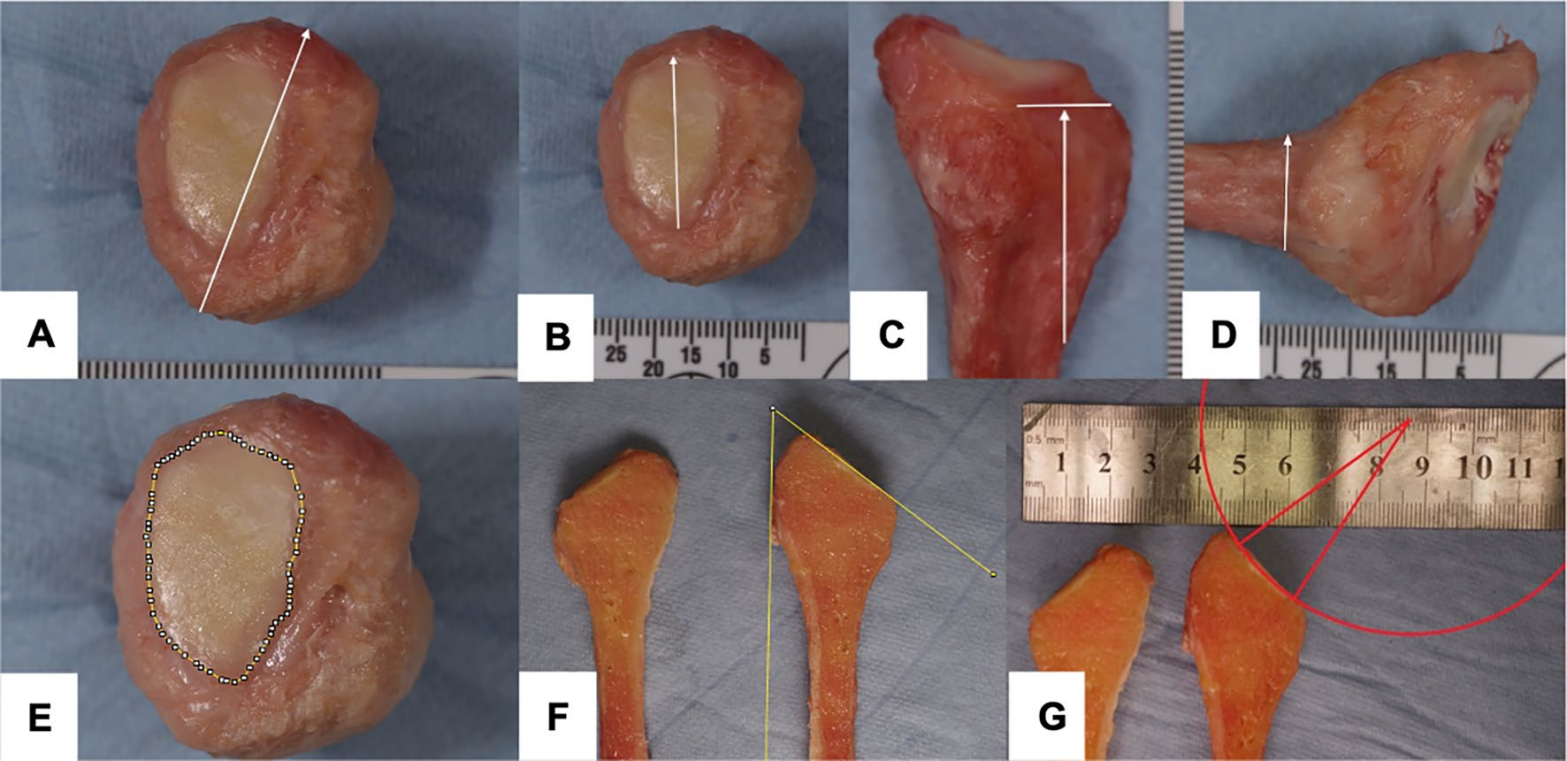
The anatomic fibular head measurements are shown: bone diameter (**A**), articular surface diameter (**B**), head height (**C**), neck diameter (**D**), articular surface area (**E**), angulation of the articular surface (**F**), and articular surface radius (**G**)

**Fig. 3:**
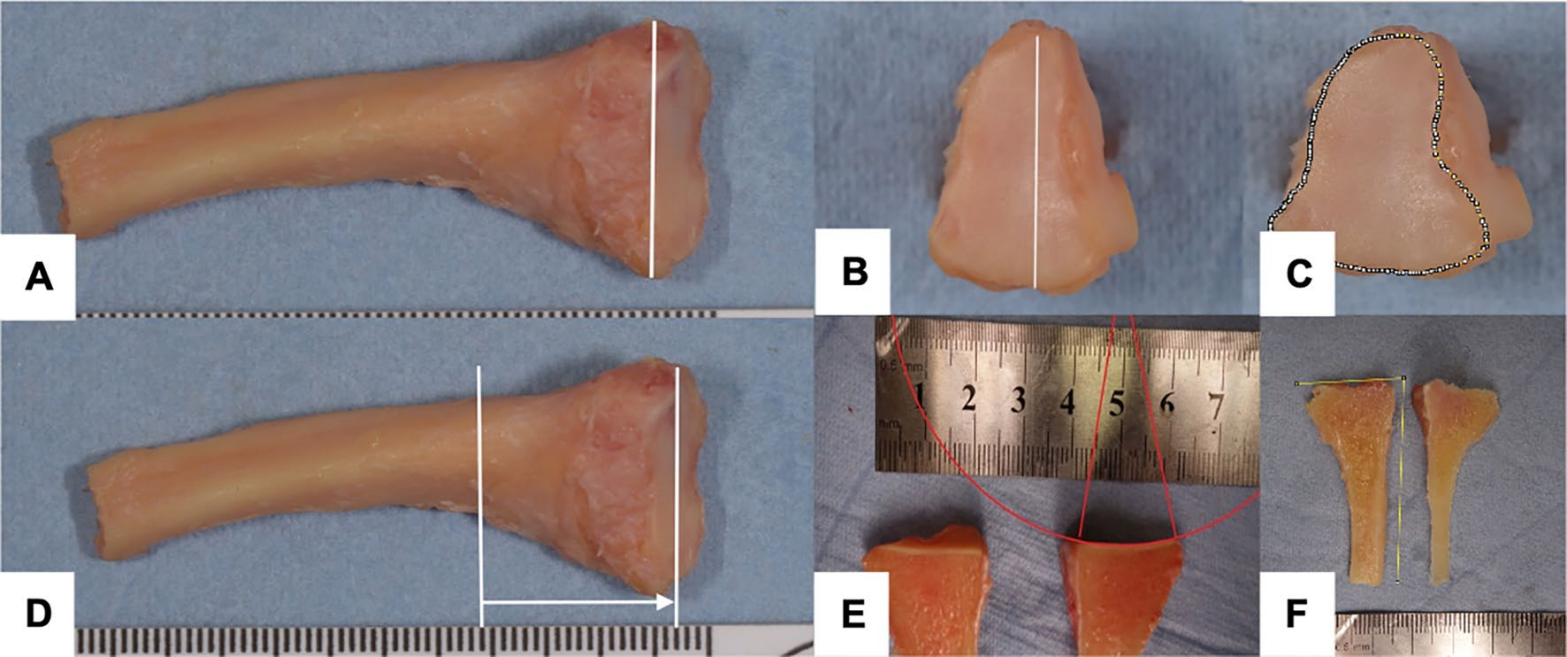
The anatomic second metatarsal base measurements are shown: bone diameter (**A**), articular surface diameter (**B**), articular surface area (**C**), head height (**D**), articular surface radius (**E**), and angulation of the articular surface (**F**)

The following parameters were measured on the ipsi- and contralateral radial head (Fig. 1), fibular head (Fig. 2) and the second metatarsal base (Fig. 3) using appropriately selected axial, sagittal and coronar images: bone diameter (minimum, maximum); articular surface diameter (minimum, maximum), head height, metaphyseal (neck) diameter (minimum, maximum), and articular surface radius.

Afterwards, all soft tissue was removed from the distal humerus and the proximal radius of the upper extremities as well as from the proximal fibula and the second metatarsal bone of the lower extremities. Thereafter, the extracted bones were cut in half following a longitudinal plane through the axis along the maximum articular surface diameter for evaluation of the articular surface radius. Each dissection phase was photographed in a standardized fashion for further evaluation. All measurements previously performed in the CT images were repeated by direct caliper-measurements (accuracy 0.05 mm) of all specimens. Total articular surface area, articular surface radius, and angulation of the articular surfaces (facet) to the longitudinal axis were measured digitally on the photographs using ImageJ (https://imagej.nih.gov/ij/index.html).

### Statistical analysis

The different specimens were compared among each other with regards to their anatomic morphometrics. Mean, standard deviation, minimum and maximum values were computed for each metric variable. A paired *t*-test was applied for all metric variables in comparisons between the three bones. An unpaired *t*-test was applied to detect side differences. Pearson’s correlation coefficient was used to evaluate anatomical measurements and CT evaluation. *R*-values were interpreted following Cohen’s definition (19). *R*-values smaller than 0.1 show no correlation, between 0.1 and 0.3 weak correlations and higher than 0.5 strong correlations. *P*-values smaller than 0.05 were deemed significant. Bonferroni-correction was applied due to multiple testing; the *p* value was thus adjusted to 0.0021 (24 tests). A second rater evaluated an initial series of photographic and CT measurements, and an ICC was established. Following the definition of Cicchetti (20), ICC was interpreted thus: 0.75–1.00 excellent; 0.60–0.74 good; 0.40–0.59 fair; < 0.40 poor agreement.

## Results

Upper and lower extremities of four female and seven male body donors were used (age 78.5 ± 11.0 years). No significant differences between sides were detected (*p* = 0.075–0.991), thus no distinction between right or left side was made for all further comparisons. Descriptive data of all measurements may be found in Table 1.

**Table 1:** Descriptive data of all measurements including statistical correlation between direct measurements and CT evaluation (Bonferroni-correction to 0.0021 was applied)

	Minimum		*R* (*p*-value)	Maximum			*R* (*p*-value)
	Direct measurement	CT		Direct measurement	CT		
Radius head diameter (mm)	23.2 ± 2.1 (19–26.5)	23.1 ± 2.2 (18.7–26.7)	0.926 (< 0.001)	24.9 ± 2.1 (20.5–28)	24.3 ± 2.1 (19.6–27.7)		0.974 (< 0.001)
Fibula head diameter (mm)	28.9 ± 3.1 (24–35)	29.3 ± 2.6 (25.4–33.6)	0.921 (< 0.001)	32.1 ± 3.1 (25–37)	29.6 ± 3.4 (21.9–36.6)		0.852 (< 0.001)
2^nd^ metatarsal base diameter (mm)	17.9 ± 2.1 (15–22.5)	17.8 ± 1.7 (14.8–22.3)	0.857 (< 0.001)	22.9 ± 2.1 (19–27)	23.8 ± 2.0 (19.8–27.75)		0.841 (< 0.001)
Radius head articular surface diameter (mm)	17.1 ± 1.9 (13.5–21)	16.9 ± 1.6 (14.3–20.1)	0.848 (< 0.001)	18.6 ± 1.9 (14–21)	18.0 ± 1.4 (15–20.6)		0.760 (< 0.001)
Fibula head articular surface diameter (mm)	14.7 ± 1.4 (12–17)	13.9 ± 2.1 (10.6–18.5)	0.508 (0.022)*	17.6 ± 2.1 (15–22)	17.6 ± 2.7 (11.2–21.4)		0.625 (0.002)
2^nd^ metatarsal articular surface diameter (mm)	8.4 ± 0.9 (6.5–10)	8.1 ± 1.4 (5.71–10.41)	0.846 (< 0.001)	20.4 ± 1.9 (16.5–25)	20.8 ± 1.9 (16.3–24.6)		0.870 (< 0.001)
Radius neck diameter (mm)	13.9 ± 1.4 (11–16)	13.8 ± 1.4 (10.6–16.2)	0.841 (< 0.001)	13.7 ± 1.6 (10.5–16)	14.3 ± 1.6 (10.8–16.5)		0.794 (< 0.001)
Fibula neck diameter (mm)	13.0 ± 2.0 (10–19)	13.3 ± 1.7 (10.5–17.3)	0.171 (0.448)*	12.8 ± 1.8 (10–16)	13.1 ± 2.1 (10.2–17.3)		0.614 (0.003)*
2nd metatarsal proximal metaphysis diameter (mm)	8.8 ± 1.3 (6–11)	8.9 ± 1.1 (7.2–11.4)	0.720 (< 0.001)	9.3 ± 1.3 (8–13)	10.0 ± 1.1 (8.43–12.4)		0.242 (0.279)*
	Direct measurement		CT			*R* (*p* value)	
Radius head height (mm)	12.3 ± 0.9 (11–14)		13.2 ± 0.9 (11.6–15)			0.484 (0.022)*	
Fibula head height (mm)	17.7 ± 1.4 (15–20)		23.6 ± 2.2 (19.1–27.7)			0.483 (0.031)*	
2nd metatarsal base height (mm)	17.1 ± 1.9 (13.5–20)		19.1 ± 2.5 (13.2–23.6)			0.729 (< 0.001)	
	Photograph-evaluation		CT			*R* (*p* value)	
Radius articular facet radius (mm)	21.1 ± 3.7 (15.5–30.4)		18.7 ± 3.9 (14.5–30.35)			0.805 (< 0.001)	
Fibula articular facet radius (mm)	33.4 ± 10.9 (16.65–57.55)		28.5 ± 8.9 (14.0–44.6)			0.936 (< 0.001)	
2nd metatarsal articular facet radius (mm)	44.4 ± 14.9 (24.5–79.5)		39.8 ± 12.4 (17.45–61.5)			0.755 (< 0.001)	
	Photograph-evaluation						
Radius proximal articular surface area (mm^2^)	458.4 ± 83.5 (299.6–591.2)						
Fibula articular surface area (mm^2^)	196.0 ± 44.0 (132.4–261.9)						
2nd metatarsal base articular surface area (mm^2^)	194.4 ± 36.2 (115.1–253)						
Radius articular facet angulation (°)	93.0 ± 4.0 (86.26–100.7)						
Fibula articular facet angulation (°)	61.1 ± 8.7 (44.5–80.7)						
2nd metatarsal articular facet angulation (°)	88.4 ± 4.1 (81.1–98.3)						

^*^No statistically significant correlation

Results were grouped into three morphologically important aspects: Facet angulation, morphology of the articular facet and morphology of the neck. A summary of all statistical comparisons including mean differences between the morphometrics of the three bones can be found in Table 2.

**Table 2:** Statistical comparison between the morphometrics of radius, fibula and second metatarsal (Bonferroni-correction to 0.0021 was applied)

Radius vs	Articular facet angulation (°)	Minimum head diameter (mm)	Maximum head diameter (mm)	Head height (mm)	Minimum articular surface diameter (mm)	Maximum articular surface diameter (mm)	Articular facet radius (mm)	Proximal articular surfaces area (mm^2^)	Minimum neck diameter (mm)	Maximum neck diameter (mm)
	Photograph	Direct	Direct	Direct	Direct	Direct	Photograph	Photograph	Direct	Direct
Fibula										
Mean difference ± standard deviation	31.9 ± 8.9	− 5.8 ± 2.2	− 7.2 ± 2.2	− 5.3 ± 1.3	2.4 ± 2.5	*1.0* ± *3.2*	− 11.9 ± 12.6	262.4 ± 89.0	0.9 ± 2.4	0.9 ± 2.3
* p* value	< 0.001	< 0.001	< 0.001	< 0.001	< 0.001	*0.151*	< 0.001	< 0.001	0.076	0.120
		CT	CT	CT	CT	CT	CT		CT	CT
Mean difference ± standard deviation		− 6.2 ± 2.4	− 5.3 ± 2.3	− 10.5 ± 2.1	2.9 ± 2.1	*0.4* ± *2.6*	− 9.8 ± 10.2		0.5 ± 2.1	1.2 ± 2.4
* p* value		< 0.001	< 0.001	< 0.001	< 0.001	*0.490*	< 0.001		0.276	0.023
2nd metatarsal										
Mean difference ± standard deviation	4.7 ± 6.6	5.2 ± 2.3	2.1 ± 2.5	− 4.8 ± 1.8	3.2 ± 1.7	− 1.8 ± 1.7	− 23.0 ± 15.0	264.0 ± 63.8	3.0 ± 1.4	0.2 ± 2.3
* p* value	0.005	< 0.001	0.001	< 0.001	< 0.001	< 0.001	< 0.001	< 0.001	< 0.001	0.745
		CT	CT	CT	CT	CT	CT		CT	CT
Mean difference ± standard deviation		5.3 ± 1.5	*0.5* ± *1.9*	− 5.9 ± 2.3	3.8 ± 1.8	− 2.7 ± 1.8	− 21.1 ± 12.6		2.3 ± 1.3	0.1 ± 1.8
* p* value		< 0.001	*0.198*	< 0.001	< 0.001	< 0.001	< 0.001		< 0.001	0.761

Marked in italics are the non-significant results

### Articular facet angulation

The facet angulation of the proximal radius and the proximal fibula differed significantly (*p* < 0.001), whereas the comparison of the facet angulation between the proximal radius and the second metatarsal base showed statistically similar values (*p* = 0.005).

### Morphology of the head and articular facet

Looking at the metrics of the radial head, only the comparison of the maximum head diameter in CT evaluation of the proximal radius and the second base metatarsal provided statistically similar values (*p* = 0.198). The minimum head diameter of the proximal second metatarsal and both measures of the fibular head as well as the height of the head in all bones differed significantly (*p* values all < 0.001).

Comparing the articular facets of the three bones, statistically similar values were found in the maximum articular surface diameter (direct, *p* = 0.151; CT, *p* = 0.490) performing direct measurements as well as CT evaluation in the proximal radius and the proximal fibula. The minimum articular surface diameter as well as facet radius and proximal articular surface area differed significantly (*p* values all < 0.001). The comparison between proximal radial and proximal second metatarsal articular facet radius provided statistically significant differences (*p* < 0.001).

### Morphology of the neck

Comparing the proximal radius and the proximal fibula, statistically similar values were found in the maximum (direct, *p* = 0.120; CT, *p* = 0.023) as well as minimum (direct, *p* = 0.076; CT, *p* = 0.276) measures of the neck diameter performing direct measurements as well as CT evaluation. Comparing the proximal radius and the proximal second metatarsal, statistically similar values were only found in the maximum neck diameter performing direct measurements (*p* = 0.745) as well as CT evaluation (*p* = 0.761).

### Interrater evaluation

Interrater evaluation showed an excellent agreement between the two raters (0.989–0.997).

## Discussion

The aim of this study was to compare anatomic details of the fibular head and second metatarsal as potential donors for an autologous radial head replacement. Introducing a vascularized autologous bone graft would be beneficial for the patients’ outcome as it would be an option to maximize usage of the elbow joint with minimal joint alteration. However, as the results of this study confirm (Table 3), no ideal autologous replacement of the proximal radius exist yet (21–23) and the challenge in this quest is rather to find a graft with the largest overlap in the most important morphometric parameters.

**Table 3:** Summary of anatomic criteria met by investigated donor bones (minimum and maximum measures were summarized as one characteristic)

	Proximal fibula	Proximal second metatarsal
Characteristics important for humeroradial articulation (4): articular surface diameter, articular surface area, facet radius, facet angulation	1/4	1/4
Characteristics important for radioulnar articulation (3): head diameter, head height, neck diameter	1/3	2/3
Summary	**2/7**	**3/7**

Apart from the medial collateral ligament, the radius is a major restraint against valgus stress, and—together with the coronoid process—also stabilizes against posterior rotational instability, which is why especially in the adolescent population, the preservation of the biomechanical role of the radius plays a large role in the therapy of radial head/neck fractures. (13, 16, 17, 24–27). In cases of radial head degeneration after trauma, resection of the head or implant replacement are the most frequent reported options left. However, resection of the head of the radius leads to an isolated force distribution through the ulna, which then may lead to the development of progressive ulnohumeral osteoarthritis despite a good clinical outcome (12). This is most often combined with a proximal migration of the radius as well as increased ulnar variance, an associated proximal radioulnar impingement and valgus as well as posterior rotational instability associated with persistent overall elbow instability and redislocations of the joint. (12, 18, 28–31) But also implant replacement of the head of the radius is not a viable option as long-term survival of the implant—which is an important factor in treating adolescents—is short compared to other joints and complications, such as loosening, wear and associated capitellar cartilage erosion, occur often (32–35). Recently, Schnetzke et al. (8) reported a rather high complication and revision rate in patients with a monopolar radial head replacement with an implant survival rate of 75.1% at 18 years after surgery with the highest failure rate in first the postoperative year.

### Proximal fibular bone graft

The fibula is a widely used bone for autologous grafts in defects throughout the body. Most often, the diaphyseal part is used and but in some cases, also proximal fibular bone grafts are described for repair of lateral malleolus or the distal radius (36, 37).

Our measurements revealed that, although in some parameters differences (neck diameter, articular surface diameter) were subjectively rather small (approx. 1 mm), the majority of values differed significantly between the radius and fibular head (>2.5 mm) (Table 2). This may be a first argument against a possible autologous transplantation of the fibular head replacing the resected radial head. Another point that may be detrimental for such a transplantation is the fact that the articular surface of the fibular head is not entirely covered by cartilage, thus leading to a large bone-cartilage interface especially between the fibular head and the radial notch of the proximal ulna. Additionally, the angulation of the articular surface is an important factor to include. The angle between the proximal articular surface of the radius and the longitudinal axis of the radius is approximately 90° (93.05±4.01), the angle between the proximal articular surface of the fibula and the longitudinal axis of the fibula is approximately 60° (61.07±8.65), thus showing a difference in angulation of 30° which leads to a biomechanically unstable interaction between the humerus and the radius with a potential of dislocation depending on the alignment of the graft. Furthermore, the variability of the angulation of the proximal articular facet of the fibula is quite high with possible angles between 10° and 90° (38).

### Second metatarsal base bone graft

The second metatarsal bone has also been described as a free bone graft for a long time and especially the distal part has since been used as a graft for reconstruction of the temporomandibular joint, the mandible, the ulnar head, the lateral malleolus and the scaphoid bone (39–44). Less frequent, the metatarsal base has been used. Del Piñal et al. (45) have described a surgical technique for reconstruction of the distal radius using the base of the second or third metatarsal bone.

With regards to transplantation of the second metatarsal, the maximum neck diameter and articular surface angulation would be arguments for such an autologous surgical procedure. However, several other measurements do definitely provide not enough similarities to make this bone a clear, considerable option, although the differences, contrary to the proximal fibula, are rather small in comparison (see Table 2). Opposed to the fibular head, a lateral (intermetatarsal) articular surface of the second metatarsal exists. But it is not long and rounded enough to provide sufficient support for the proximal radioulnar articulation. Moreover, the shape of the second metatarsal base is rather different compared to the radius. Despite these largely statistically significant differences, this does not necessarily mean that an autologous transplantation is to be completely rejected because minor (significant) differences might still be clinically acceptable. In addition, the angulation of the proximal articular surface of the second metatarsal bone is similar to the angulation of the radius with less variability (see Table 1), leading to a possibly more stable articulation with the humeral capitulum.

Apart from morphometric disadvantages of the second metatarsal base bone graft, one has also to keep in mind some further disadvantages for its use, e.g., one creates an isolated iatrogenic Lisfranc ligament injury when harvesting the bone, which consequence remains unclear until now (46–48).

### Graft choice for radial head replacement

A comparison of our data with the current literature and thus an objective graft recommendation is very difficult because only one article proposing an autologous bone graft for radial head replacement currently exists to the best of our knowledge. Han et al. (48) described an autologous second metatarsal transfer to the head of the radius in five patients. The authors used 1.5–2-cm non-vascularized second metatarsal base grafts to reconstruct the proximal radius. The presented postoperative results were good to excellent in the short-term (mean follow-up 44.8 months) with regards to elbow range-of-motion and donor site problems. The authors facilitated transplantation by shaving parts of the second metatarsal base to accommodate its placement into this joint area and imitate the shape of the head of the radius. However, objective parameters for graft selection are missing.

As described before, two important articulations have to be considered when replacing the proximal radius. In the humeroradial joint, the articular contact area and the lateral collateral ligament have to be taut similar to the anatomical situation to maximize valgus and posterior rotational stability of the elbow joint. Here, graft length, size of the proximal articular facet and facet angulation would be important parameters to consider. Graft length depends on the defect to repair and is easily facilitated through both the proximal fibula and the proximal second metatarsal. The size of the articular facet is in both the fibula and the second metatarsal significantly smaller than the facet of the radius and concerning the facet angulation, only the angulation of the second metatarsal bone is rather similar to the radius (see Table 2). In the proximal radioulnar joint, the size of the head and neck play an important role in the interaction with the annular ligament and the radial notch of the ulna to facilitate pronation-supination movement. Here, the head or base size, respectively, of the proposed grafts do not seem to meet the criteria but the size of both necks seem to. However, both grafts are no perfect fit for the radial notch of the ulna (see Table 3).

To compensate for the suboptimal fit of the second metatarsal base to the radial notch of the ulna, an additional anconeus interposition (49), Achilles tendon interposition (9) or corium interposition (11) could be options to optimize the radioulnar movability. Alternatively, a costochondral graft has been described especially for bone replacement in children. It could have benefits due to its compatibility, applicableness, processability and rarity of additional functional damage to the patient. Additionally, this graft has shown the potential of growth, which is of additional advantage in children. However, this growth potential may also be of disadvantage as unpredictable growth with graft hyperplasia has been reported (50).

Some limitations have to be taken into account when interpreting the presented results. As common in anatomical studies, the age of the body donors is significantly higher than the intended target group. As the morphometric comparison was applied between the different characteristics of bones of the same donors, the significance of differences is still applicable, although the exact size is not. Also, some parameters may be influenced by high anatomical variability, which would need a larger number of specimens to meet the necessary sample size. Nonetheless, the presented results reflect a viable cross section sufficient to answer the initial research query. Further limitations may be attributed to the clinical applicability. As pediatric radial head fractures may lead to post-traumatic axial deviation, replacement of the radial head with an autologous graft may therefore only be considered in selected cases. It must also be considered whether one accepts the donor morbidity for a questionable gain in function at the elbow.

In summary, neither the proximal fibula nor the base of the second metatarsal are an ideal bone graft for replacement of the head of the radius. Both bones do have several unsuitable parameters, while the remainder is somewhat moderately useful. While the fibular head does exhibit increased absolute values compared to the proximal radius, those of the second metatarsal are overall smaller than the head of the radius. Considering our predefined, most relevant anatomic criteria, the base of the second metatarsal might be a bit more suitable as a potential donor, especially as the angulation of the proximal articular facet is similar to the angulation of the radius. To gain further knowledge into this topic, biomechanical studies are needed.
